# An In Vitro Study on the Role of Cellulases and Xylanases of *Bacillus subtilis* in Dairy Cattle Nutrition

**DOI:** 10.3390/microorganisms12020300

**Published:** 2024-01-30

**Authors:** Valeria Bontà, Marco Battelli, Erlinda Rama, Michela Casanova, Lorenzo Pasotti, Gianluca Galassi, Stefania Colombini, Cinzia Calvio

**Affiliations:** 1 Laboratories of Genetics and Microbiology, Department of Biology and Biotechnology, University of Pavia, Via Ferrata 9, 27100 Pavia, Italy; 2 Department of Agricultural and Environmental Sciences, University of Milan, Via Celoria 2, 20133 Milan, Italy; marco.battelli@unimi.it (M.B.); gianluca.galassi@unimi.it (G.G.); stefania.colombini@unimi.it (S.C.); 3 Department of Electrical, Computer and Biomedical Engineering, University of Pavia, Via Ferrata 5, 27100 Pavia, Italylorenzo.pasotti@unipv.it (L.P.)

**Keywords:** ruminal fermentation, animal probiotics, direct-fed microbials, fiber degradability, gas production

## Abstract

The administration of *Bacilli* to dairy cows exerts beneficial effects on dry matter intake, lactation performance, and milk composition, but the rationale behind their efficacy is still poorly understood. In this work, we sought to establish whether cellulases and xylanases, among the enzymes secreted by *B. subtilis*, are involved in the positive effect exerted by *Bacilli* on ruminal performance. We took advantage of two isogenic *B. subtilis* strains, only differing in the secretion levels of those two enzymes. A multi-factorial study was conducted in which eight feed ingredients were treated in vitro, using ruminal fluid from cannulated cows, with cultures of the two strains conveniently grown in a growth medium based on inexpensive waste. Feed degradability and gas production were assessed. Fiber degradability was 10% higher (*p* < 0.001) in feeds treated with the enzyme-overexpressing strain than in the untreated control, while the non-overexpressing strain provided a 5% increase. The benefit of the fibrolytic enzymes was maximal for maize silage, the most recalcitrant feed. Gas production also correlated with the amount of enzymes applied (*p* < 0.05). Our results revealed that *B. subtilis* cellulases and xylanases effectively contribute to improving forage quality, justifying the use of Bacilli as direct-fed microbials to increase animal productivity.

## 1. Introduction

Current challenges for the livestock sector are the identification of technologies able to boost animal performance through sustainable farming systems [1,2]. Sustainability can be met by raising animal productivity through the improvement of the nutritional properties of current forages or sub-optimal feeds, thereby avoiding further exploitation of natural resources. Forage fibers are mainly constituted by cellulose and hemicellulose and animal productivity has been often correlated with their degradability by the rumen microorganisms [2,3]. A higher fiber degradability translates into a lower amount of feed necessary to produce a unit of meat or milk, reducing the impact of animal production on environmental resources. Another non-negligible advantage of incrementing fiber degradability is the possibility of using lower nutritional biomasses that, differently from grains, are not in competition with human nutrition.

To increase the fiber surface area accessible to rumen microbial activity, several forage treatments have been considered, ranging from physicochemical approaches, e.g., treatments with strong alkalis, ammonium, urea, or steam [4,5], to more environmentally friendly processes based on the application of exogenous hydrolytic enzymes to deconstruct the lignocellulosic biomass [6,7]. However, the latter strategy has a heavy impact on farm profitability due to the high costs of industrial enzymes and, importantly, enzymatic activity is often rapidly lost within the rumen environment [8].

The supplementation of probiotic microorganisms, also known as direct-fed microbials (DFM), which can directly produce degradative enzymes into forages and diets, may overcome such limits, guaranteeing continuous secretion and turnover of hydrolytic enzymes to deconstruct the biomass, eventually facilitating its fermentation in the rumen [9].

Numerous microorganisms are known to secrete hydrolytic enzymes that attack the lignocellulosic matrix of vegetable materials, mainly represented by fungi and soil bacteria [10]. One of the best-characterized bacterial species known to produce degradative enzymes is *Bacillus subtilis*. Its attractiveness not only derives from its propensity to secrete massive amounts of enzymes directly into the growth medium (up to 20–25 g/L) but also from the ease of cultivation and its recognized safety in human and animal nutrition [11,12]. Dwelling mainly in the upper layers of soil and in the plant rhizosphere, the *B. subtilis* genome has evolutionarily accumulated a large set of degradative enzymes [13]. This species, listed in the Qualified Presumption of Safety (QPS)-recommended microorganisms intentionally added to food or feed [14], was shown to colonize the gastrointestinal tract of mammals and monogastric animals in the form of vegetative cells (spores) or by forming biofilms, thereby acting as a probiotic [15,16,17,18].

*Bacillus*-based DFMs are already in use in several monogastric animal species, with positive impacts on nutrient absorption and in immune response ascribed to the alteration of the microbial ecology in the digestive tract [17,18]. *B. subtilis*-based DFM have also been applied to dairy cattle, in which they consistently exerted a positive influence on ruminal fermentation, growth, lactation performance, and milk composition [19,20,21,22,23,24]. Yet, the bacterial functions that are at the basis of such beneficial effect still await elucidation [9,16]. In line with its soil origin and its high enzymatic ability [12,13], a role in degradation of the vegetable fiber contained in several types of feeds has been attributed to *B. subtilis* through in vitro studies [25,26,27,28]. Unfortunately, the consistency of the results was often compromised by the use of different, and often poorly characterized, bacterial strains.

It is still unclear which enzymatic activities, among those commonly found in the genetic repertoire of *Bacilli* [29], are crucial players in feeds fermentation in the ruminal environment. This point can only be addressed using in vitro analyses, which can provide specific and reproducible data rapidly and conveniently [3]. Particularly important are in vitro rumen fermentation assays that allow the assessment of the degradability of the neutral detergent fiber content of feeds (NDFd) and the ruminal gas production (GP), which is an indirect measurement of rumen microbial fermentation; GP is often used to complement NDFd analyses for the evaluation of the nutritive value of feeds as it positively correlates with the degradability and nutritional value of forages [3,25,26,27,28,30].

From a common laboratory strain of *B. subtilis*, we recently derived an isogenic strain in which the production of endogenous cellulase and xylanase was optimized [31,32], while the synthesis of additional hydrolytic enzymes, such as amylase, pullulanase, and proteases, also involved in feed degradability, remained unaltered.

Taking advantage of the very specific differences between the engineered and the parental strain, in this work, we sought to determine whether the secretion of these crucial enzymes for fiber degradation contributes to feeds fermentation in the ruminal environment. To this aim, fresh cultures of the two *B. subtilis* strains (parent vs. optimized strain) were directly applied to several types of common cattle feeds. Moreover, to preserve the affordability of forage treatment, *B. subtilis* cultures were conveniently grown in a salt medium in which the carbon source derived from minimally processed rice straw, which is a low-cost, annually renewable, agricultural waste and the third most abundant world biomass, not in competition with the food or feed industry [33]. NDFd and GP were experimentally measured to evaluate the contribution of cellulolytic and xylanolytic activities of *B. subtilis*. This study provides a scientific rationale for the effect of *Bacilli* as cattle probiotics and might be relevant in determining which characteristics should be considered valuable in the selection of DFM strains.

## 2. Materials and Methods

### 2.1. Bacillus subtilis Strains

*Bacillus subtilis* strains used in this study are the wild-type (WT) PB5700 strain and its engineered version PB5703 (PB2OPT). PB5700 is a spontaneous *swrA*^+^ derivative of the common laboratory strain JH642 strain [34] (GenBank accession no. CM000489.1) in which the auxotrophies caused by the *trpC2 pheA1* mutations have been cured as described [32]. PB2OPT was derived from PB5700 by genetic optimization without the introduction of any exogenous DNA sequence and is similar to strain OS58 [32]. A patent application is under preparation for this strain; for this reason, additional details on the engineered strain are herein undisclosed.

### 2.2. Bacterial Medium Preparation and Its Characterization

Open-air-dried rice (*Oryza sativa* L.) straw was collected from two local farmers in the Pavia area (in the northwestern part of Italy) and stored in burlap sacks. Upon use, straw was minced using a kitchen blender (Moulinex AY45 Easy Power Blender, Faucogney-et-la-Mer, France) and dried to constant weight in a 60 °C oven. One liter of tap water was added to 25 g of straw and the suspension was vigorously stirred at room temperature for 2 h with a magnetic stirrer. The straw was removed through a 1 mm mesh strainer covered with two sheets of gauze. The washing liquid (WL) was collected and subjected to low-pressure sterilization (5 psi/~0.35 bar), to avoid sugar caramelization, for 20 min and, then, stored at −20 °C.

To quantify the sugars extracted, 300 µL of WL was brought to a final volume of 500 µL with 50 mM Na–acetate buffer pH 5 and incubated at 50 °C for 64 h with shaking, either alone or in the presence of 22 µL–enzymes cocktail constituted by 1U cellulase (product no. 22178, MilliporeSigma, Burlington, VT, USA), 1U beta-glucosidase (product no. 49290, MilliporeSigma), 1U Xylanase (product no. X2629, MilliporeSigma), and 0.013 U cellobiohydrolase I (product no. E6412, MilliporeSigma). A mock reaction was carried out with the enzymatic cocktail in the absence of substrate.

After incubation, samples were centrifuged at 16,873× *g* at room temperature for 5 min, filtered through sterile 0.2 µm polyethersulfone (PES) filters, and stored at −20 °C until HPLC analysis. Twenty-five microliter samples were injected through an automatic injector and analyzed in an LC-2000 HPLC system (Jasco, Tokyo, Japan) equipped with a Supelco C-610H 30 cm × 7.8 mm column (59320-U, Sigma-Aldrich, Saint Louis, MI, USA) and a RID 10A detector (Shimadzu, Tokyo, Japan). For chromatography, 0.1% H_3_PO_4_ was used as the mobile phase with 0.5 mL/min flow rate. Column temperature was kept at 30 °C. Chromatograms were analyzed by the ChromNAV 2.0 software (Jasco, Tokyo, Japan). The column was previously calibrated with standard solutions of glucose, xylose, galactose, mannose, arabinose, and cellobiose, serially diluted in the mobile phase to prepare calibration curves based on at least three replicates for each sugar. The lower limit of quantification for all sugars was 0.3 g/L.

Xylose, galactose, and mannose showed the same retention time and the same concentration-peak area calibration lines, thereby enabling the precise quantification of their sum. However, since the rice straw content in galactose and mannose is negligible compared with the one of xylose, we assumed that the concentration of co-eluted xylose, galactose, and mannose detected was only due to xylose [35,36]. No chromatogram peaks corresponding to arabinose or cellobiose were detected. Background sugars present in the enzymatic cocktail were subtracted from each sample. For control samples with values below the limit of quantification (LOQ) of the calibration curve (0.3 mg/mL), the LOQ/2 value (i.e., 0.15 mg/mL) was used as background [37]. Sugar quantification values are the mean of two independent experiments.

### 2.3. Bacillus subtilis Cultures

The growth medium was prepared by adding to WL the following components: 7 g/L K_2_HPO_4_; 2 g/L KH_2_PO_4_; 0.5 g/L Na citrate; 0.1 g/L MgSO_4_; 1 g (NH_4_)_2_SO_4_; and 1 g/L asparagine. PB5700 and PB2OPT spores were revitalized overnight on LB plates. A few single colonies were inoculated in 3 mL of Antibiotic Medium 3 (BD-Difco, Franklin Lakes, NJ, USA) and grown at 37 °C with orbital shaking until culture density was appreciable by visual inspection. Pre-cultures were used to inoculate 20 mL of fresh Antibiotic Medium 3 containing 5 mg/mL glucose which was grown at 37 °C overnight. Fermentation was carried out in flasks for 24 h in the WL-based medium at 37 °C with 150 rpm agitation, starting with an optical density at 600 nm (OD_600_) of 0.2. Each fermentation was independently repeated at least three times.

### 2.4. Cellulase and Xylanase Activity Assays

At the end of 24 h fermentations, cellulase (endo-1,4-β-glucanase; EC 3.2.1.4) and endo-xylanase (endo-1,4-β-xylanase; EC 3.2.1.8) activities were assayed in the culture supernatants, after acidification, using the CellG5 and XylX6 kits (Megazyme, Wicklow, Ireland) as previously described [38].

### 2.5. In Vitro Analyses

The effect of the enzymes produced by PB5700 and PB2OPT on the degradability of feeds was analyzed using two different in vitro incubation methods aimed to evaluate ruminal degradability of NDFd and GP. For both methods, ruminal fluid was collected from three rumen-cannulated cows (non-lactating Holstein–Friesian). The cows were fed a total mixed ration (TMR) composed of 66% hay from permanent grass and 34% commercial concentrate formulation (composed of soybean meal, corn meal, molasses cane, wheat middlings, wheat grain, and a mineral–vitamin mix) twice daily to achieve a dry matter intake (DMI) of 8 kg/d. Rumen liquor was collected two hours after the morning feeding, immediately strained through four layers of cheesecloth into a pre-warmed (39 °C) flask, flushed with CO_2_, and used within one hour from sampling. The donor animals were handled as outlined by the Directive 2010/63/EU on animal welfare for experimental animals and all animal procedures were conducted under the approval of the University of Milan Ethics Committee for animal use and care and with the authorization of the Italian Ministry of Health (authorization no. 904/2016-PR).

For both NDFd and GP experiments, three different treatments were analyzed: (i) the control treatment, constituted by the WL-based growth medium, in the absence of bacteria; (ii) the treatment, constituted by PB5700 culture; and (iii) the treatment, constituted by PB2OPT culture.

The incubations were conducted on 8 different feed ingredients collected in Northern Italy (Lombardy region), representing a wide range of NDF concentrations: 2 different meadow hays (on average, 53.2 ± 4.1% on DM), 2 different alfalfa hays (on average, 54.3 ± 6.9% on DM), 2 different corn silages (on average, 45.8 ± 9.1% on DM), and 2 different TMRs for lactating cows (on average, 35.9 ± 1.1% on DM). All feeds were dried at 60 °C for 48 h in a forced-air oven and ground to pass a 1 mm Fritsch mill (Fritsch Pulverisette, Idar-Oberstein, Germany). All the experiments (three treatments × 8 feed ingredients) were independently repeated three times (three incubation runs for each method).

#### 2.5.1. In Vitro NDF Degradability

The NDF degradability was determined at 48 h using the Ankom Daisy II incubator (Ankom Technology, Macedon, NY, USA) as previously reported [39] and modified as follows: each jar, containing 250 mg of each feed weighted in triplicate in Ankom F57 bags, was inoculated with 665 mL of fresh bacterial culture from one of the three experimental treatments (control, PB5700, and PB2OPT), 133 mL of salt mix solution (90 g/L KH_2_PO_4_; 5 g/L NaCl; 5 g/L urea; 4.5 g/L MgSO_4_·7H_2_0; and 1 g/L CaCl_2_·2H_2_0), 532 mL of distilled water, 266 mL of reducing solution (Na_2_CO_3_ 15 g/L; and Na_2_S·9H_2_O 1 g/L), and 400 mL of rumen fluid. The NDF content of feed ingredients at the end of incubation was determined using the Ankom 200 fiber analyzer (Ankom Technology, Macedon, NY, USA) following the procedure reported [40].

#### 2.5.2. In Vitro Gas Production

GP was analyzed for 48 h by incubating 200 mg of each feed in individual 120 mL serum bottles for each of the three treatments (control, PB5700, and PB2OPT) as previously described [41]. Each bottle was inoculated with 30 mL of final solution made up of 8.33 mL of fresh bacterial culture, 1.67 mL of salt mix solution (described above), 6.7 mL of distilled water, 3.33 mL of reducing solution (described above), and 10 mL of rumen fluid; for the control treatment (uninoculated culture), deionized water replaced the WL fraction as, alone, the sugars contained in the WL, and not consumed in the absence of *B. subtilis*, could be fermented by the rumen bacteria developing a high level of gas. For each treatment, 2 blanks (bottles containing just the buffered rumen fluid, prepared according to the corresponding treatment but lacking the substrate feed) were also included, as a measure of the gas produced in the absence of any feed. GP was determined measuring the headspace pressure in the incubation vials [42]. The pressure was recorded after 24 and 48 h of incubation using a digital manometer (model 840082, Sper Scientific, Scottsdale, AZ, USA). The values of pressure obtained from GP experiments in vitro were converted into volume (mL) of gas produced at standard temperature (0 °C) and pressure (1 bar) using the ideal gas law. The GP of each treatment was corrected with the respective blank before statistical analyses.

At the end of the incubation, fermentation was stopped by putting all vials into an ice bath and the pH was recorded. The content of each vial was individually transferred into 50 mL Falcon tubes and centrifuged at 10,000× *g* for 15 min. After centrifugation, 5 mL and 30 mL of supernatant were sampled for subsequent volatile fatty acids (VFA) analysis as previously reported [43]. The NH_3_ concentration was determined with a Raypa NP-1500-MP Kjeldahl distiller (Raypa, Terrassa, Spain).

### 2.6. Calculations and Statistical Analyses

Data, assumed to follow a normal distribution, were statistically analyzed by the PROC MIXED procedure of SAS 9.4 (SAS Institute Inc., Cary, NC, USA), with the following model:

Y_ijk_ = μ + T_i_ + F_j_ + R_k_ + T_i_ × F_j_ + ε_ijk_

where Y_ijk_ = dependent variable, μ = overall mean, T_i_ = fixed effect of the treatment (i = 1 to 3), F_j_ = fixed effect of the feed (j = 1 to 8), R_k_ = random effect of the fermentation run (k = 1 to 3), T × F_ij_ = interaction treatment × feed, and ε_ijk_ is the random error. The least-square means were reported. For all statistical analyses, significance was declared at *p*-value (*p*) ≤ 0.05 and trends at *p* ≤ 0.10.

## 3. Results

### 3.1. B. subtilis Feed Additive Preparation

An inexpensive procedure for the production of affordable *B. subtilis*-based DFM was set up through a simplified protocol that could be carried out at the farm site. Rice straw was selected as the carbon source because it represents an inexpensive and abundant agricultural waste and contains significant amounts of water-soluble carbohydrates, i.e., fructose, sucrose, and β-1,3-1,4-glucan [44,45]. To extract those sugars through an environmentally friendly and simple process, dried rice straw was vigorously washed with tap water at room temperature. HPLC analyses of the washing liquid (WL) recovered demonstrated the presence of monomeric sugars; to verify whether complex sugars were also present in WL, enzymatic hydrolysis of the WL liquid was carried out with commercial enzymes (see Section 2, Material and Methods). As shown in Table 1, the monomeric sugars increased substantially after hydrolysis (300% and 40% for glucose and xylose, respectively), demonstrating that complex sugars could also be recovered by rice straw washing.

The non-hydrolyzed WL fraction was, thus, used as the carbon source for the growth of *Bacillus subtilis* in the low-cost medium. Two isogenic laboratory strains were used: PB5700, as the wild-type (WT) strain, and the engineered PB2OPT strain, only differing from the former for the optimized secretion of cellulases and xylanases. Both strains were able to grow in shake flask conditions on the WL fraction in a comparable manner, without the requirement of preliminary saccharification of the medium, reaching a final optical density at 600 nm (OD_600_) of 2.85 ± 0.14 for the WT and of 3.83 ± 0.14 for PB2OPT at 24 h.

**Figure 1:**
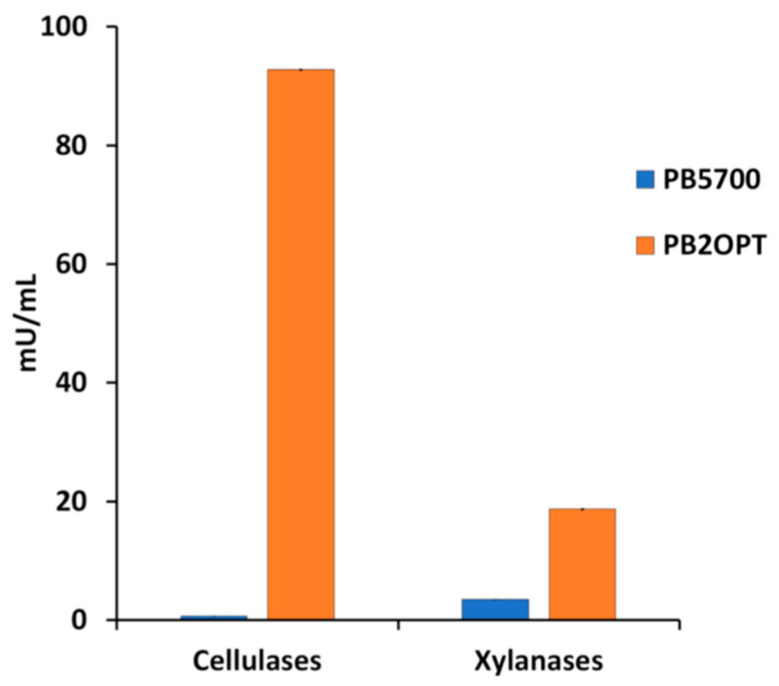
Cellulase and xylanase activities. Cellulase and xylanase activities, expressed in mU per mL of culture media, recorded in the culture supernatant of PB5700 and PB2OPT strains grown on WL medium. Values are the average of at least five independent experiments; error bars (included, although barely visible) represent the standard error of the means.

Cellulases and xylanases, released from both strains in the growth medium, were quantified at the end of the incubation. As expected, secretion of both enzymes by the optimized strain was much higher (Figure 1). Cellulase activity in the supernatant of PB2OPT was 142-fold higher than for PB5700 (92.8 ± 1.6 mU/mL vs. 0.7 ± 0.2 mU/mL, respectively). Xylanase activity was also significantly higher in PB2OPT, albeit only 5.4-fold (18.7 ± 2.2 mU/mL vs. 3.5 ± 0.4 mU/mL).

Through the simple process described, a low-cost medium was successfully obtained that supported the 24 h growth of the two strains to be used for feed treatments.

### 3.2. Feed Treatment and Fiber Degradability

Each bacterial culture was directly applied to common feed ingredients for dairy cows, such as meadow hay, alfalfa hay, maize silage, and total mixed ration (TMR) and incubated with feeds in a simulated in vitro rumen environment. Two different sources of each feed type were subjected to identical treatments. Raw cultures of the two *B. subtilis* strains, which included bacteria and the growth medium in which degradative enzymes had accumulated, were used without any preliminary enzyme or bacterial purification steps to preserve the economic affordability of the treatment. Sterile WL growth medium was used as the control treatment.

NDFd was evaluated in all the conditions described (two strains plus a control treatment, with 8 feeds). Analyzing the pool of feed ingredients as a whole, a significant increment in NDFd was observed for both strains with respect to the control and also between each other (*p* < 0.001) (Table 2). In the samples treated with the WT *B. subtilis* strain, NDFd attained 45.6%, showing a 5% increase compared to the control (43.4%). A remarkable 10% increment in NDFd (47.8%) with respect to the control was observed when the treatment was carried out with the PB2OPT strain, which secretes higher amounts of fibrolytic enzymes. The degradative enzymes present in the whole cultures at the beginning of the treatment as well as those secreted over time during the treatment may contribute to the described NDFd increase in which PB2OPT-treated feeds showed the highest degradability index.

**Figure 2:**
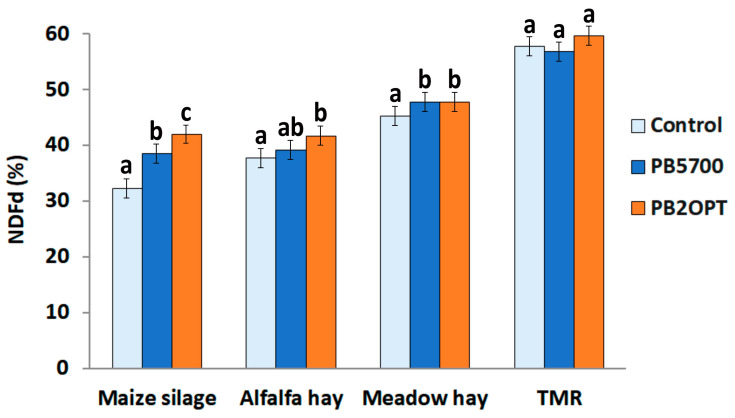
Fiber degradability of forages. Fiber degradability expressed as NDFd (%) in the different types of feed ingredients upon treatments: bars represent average values of the treatments with negative control without bacteria (light blue bars); PB5700 parent strain (blue bars), and cellu-lase/xylanase-overexpression PB2OPT strain (orange bars). Within feeds, mean values with different lettering are statistically different (*p* < 0.05).

NDFd data also revealed a significant interaction between treatment × feed (*p* = 0.028). Particularly, the high NDF content in maize silages (on average, around 45.9% in our heterogeneous samples) was significantly more sensitive to enzymatic digestion and its degradation efficiency correlated with the amount of degradative enzymes released by the strains (PB2OPT > PB5700 > control) (Figure 2); alfalfa hay showed a significant increment in NDFd only in the presence of the optimized strain, although a gradual trend was appreciable, whereas for meadow hay both strains equally contributed to the increase in NDFd as compared to the control (Figure 2). Unsurprisingly, no effect of the bacterial treatment was observed on TMR forage, characterized by a lower NDF content (on average, 34% in our samples).

### 3.3. Gas Production and Ruminal Fermentative Profile

The gas produced from in vitro ruminal fermentation of the experimental feeds upon *B. subtilis* treatment demonstrated that both strains were able to improve GP compared to the control (Table 2): regardless of the strain, gas was released more effectively in the presence of *B. subtilis*. Although differences among the two strains just showed a trend (*p* < 0.05), the treatment with the PB2OPT strain produced the highest gas volume both at 24 (+9.36% and +1.67% with respect to the control and the WT strain, respectively) and 48 h of incubation (+11.9% and +1.86% with respect to the control and the WT strain, respectively). These data confirmed that *B. subtilis*, in general, improved the degradability of the feeds. Indeed, *B. subtilis* secretes a large number of degradative enzymes in addition to cellulases and xylanases (e.g., proteases, amylases, pullulanases, pectinases, and others) which do not differ between the two strains. Therefore, the limited difference observed between the two strains could be ascribed to the fact that the enzymatic mixture produced by the WT strain is already sufficient to increase the degradation of the feeds within the 48 h incubation.

Moreover, as observed for the NDFd, also in GP, the effectiveness of the enzymatic pool is linked to the different chemical composition of the individual substrates, as shown by the interaction between treatment × feed (*p* < 0.10). The analyses of single feed ingredients indicated that for meadow hays, the feed with the highest fiber content, PB2OPT was more effective than the WT strain in improving GP (GP 48 h/g DM was, respectively, 95.3, 113, and 125 mL for control, PB5700, and PB2OPT), underlying the relevance of cellulase and xylanase activities for the fermentation of this type of substrate. The data presented in Table 2 also show that GP was higher during the first 24 h of incubation rather than in the following 24 h; this effect was most likely due to rapid fermentation of the easily degradable fractions, such as simple sugars and non-structural carbohydrates (such as starch). Remarkably, the positive effect of the *B. subtilis* treatments on GP, which was immediately appreciable (*p* < 0.001 for GP at 24 h), was maintained until the end of the incubation (*p* < 0.001 for GP at 48 h).

Concerning the fermentative profile, N-NH_3_ concentration was only affected by the feed (*p* < 0.001) and not by the treatment: it was higher for alfalfa and TMR samples (on average, 50.5 mg/dL) than for meadow and corn silage samples (45.9 mg/dL) due to their higher crude protein content. Similarly, acetic acid production was also mainly affected by the feed (*p* < 0.001), showing a lower concentration in TMR and corn silage samples (on average, 55.3% vs. 59.3% in alfalfa and meadow). There was a trend towards a lower propionic acid content (% total VFA) (*p* = 0.063) in samples treated with either PB5700 or PB2OPT (on average, 20.2%) as compared to the untreated control (21.4%). The slightly lower release of propionic acid in those samples, although not significant, can be related to a higher fiber degradability; the proportion and type of volatile fatty acids produced are, in fact, dependent on the substrate metabolized and fiber fermentation usually promotes higher acetic production and lower propionate formation.

## 4. Discussion

The analyses of the global effect of *B. subtilis* on NDFd and GP (Table 2) showed that both *B. subtilis*-based DFM enhanced the rumen function and increased feed quality, as already demonstrated by several authors both in vivo and in vitro [19,20,21,22,23,24,25,26,27,28]. The enzymes overexpression provided a 5% increase in NDFd with respect to the WT strain. Although the increment is apparently not dramatic, it is worth recalling that 1% increase in forage NDFd is associated with a 0.17 kg/d increase in DMI and 0.25 kg/d of milk produced [2].

In agreement with NDFd data, the GP also increased upon treatments. The increases in GP were slightly higher than in other work [24] but this result might be explained by the release of reducing sugars and other products caused by the addition of fibrolytic enzymes in in vitro experiments, which could both enhance rumen microbial population and, thereby, increase the amount of feedstuff digested.

Since enzymes efficacy is primarily related to the type of feed and its lignin content [2,6], the feeds selected for this study were highly diversified in chemical composition and NDF degradability. According to the Cornell net carbohydrate and protein system (CNCPS), maize silage, meadow hay, and alfalfa hay contain 3.5%, 4.5%, and 6.5%, respectively, of potentially degradable fibers. Consistent with previous findings, the sensitivity of forages to *B. subtilis* DFM (Figure 2) was inversely related to the intrinsic degradability of the feed [2]. Maize silage, which contains the most recalcitrant fiber to ruminal activity among the substrates tested (in terms of digestion rate), benefited the most from the support of bacterial enzymes. Conversely, the low NDF content (on average, 34% of the dry matter) of our TMR samples might explain the lack of efficacy of the bacterial treatment for this substrate.

Overall, the increase in NDFd observed in the present study was similar to the average increase reported by other authors [24,27,28]; small differences in quantitative performance indexes might be due to the different strains utilized and, more importantly, to the local sources of fodders. Our experiments were conducted in the Mediterranean area (Lombardia region, Italy) and the nutritive value of local forage and diets might differ from that used in other studies. Differences in feed sources may affect the NDF degradability values, partially explaining the variability between studies [6,24].

The main novelty of this work is represented by the fact that the engineered strain differed from the parental one exclusively for the overexpression of cellulases and xylanases; the comparison of the activity of the two strains offered the possibility of gaining a conclusive indication on the efficacy of the cellulases and xylanases secreted by *B. subtilis*, excluding any confounding effect. Therefore, the most important conclusion is the unambiguous demonstration that these two enzymes have a relevant role in supporting the ruminal flora in the recovery of nutrients from fibrous plant material.

By applying the same strategy, it will be possible to obtain new strains, each engineered for the overexpression of a different set of enzymes, even heterologous ones, as hypothesized by Wang and McAllister in 2002 [6], that can be challenged with different substrate feeds through simple, easily parallelizable in vitro tests. The results of these tests will help in designing ideal probiotic strains tailored for the optimal digestion of locally and/or seasonally available forage to be validated through more complex, expensive, and informative in vivo experiments. In addition, similar studies can be applied to feeds used in poultry and swine weaning, where the addition of xylanases, in particular, has been shown to increase animal health and growth performances [46].

With this objective in mind, we also designed a convenient, sustainable, and simplified production process through which *B. subtilis* feed additives can be obtained from inexpensive raw cultures grown on an abundant agricultural waste biomass such as rice straw. Following the designed procedure, feed additives could be produced on site and, possibly, be integrated into local biorefineries, creating a virtuous circular economy process.

## Figures and Tables

**Table 1:** Monomeric sugars contained in the straw washing liquid (WL) before and after enzymatic hydrolysis.

	Before Hydrolysis		After Hydrolysis	
Carbon Source	Glucose ^1^	Xylose ^1^	Glucose ^1^	Xylose ^1^
WL (mg/mL)	0.542 ± 0.012	0.500 ± 0.000	2.183 ± 0.519	0.700 ± 0.047

^1^ glucose, xylose, arabinose, galactose, mannose, and cellobiose were quantified by HPLC. Only those identified in the samples are given as mg/mL of WL. Values are the average of two independent experiments with standard deviations.

**Table 2:** Effects of the inclusion of the two strains of *B. subtilis* on fiber degradability, gas production, and fermentation parameters.

	Treatment ^1^			SE ^2^	*p*-Value ^3^		
	Control	PB5700	PB2OPT		T	Feed	T × Feed
NDFd ^4^ (%)	43.4 ^a^	45.6 ^b^	47.8 ^c^	1.30	<0.001	<0.001	0.028
GP ^5^ 0–24 h (mL/g DM)	83.3 ^a^	89.6 ^b^	91.1 ^b^	5.07	0.019	<0.001	0.099
GP 24–48 h (mL/g DM)	29.3 ^a^	32.2 ^b^	32.8 ^b^	1.56	0.016	<0.001	0.757
GP 0–48 h (mL/g DM)	113 ^a^	122 ^b^	124 ^b^	5.25	0.010	<0.001	0.217
pH	6.26	6.29	6.28	0.161	0.239	<0.001	0.392
N-NH_3_^6^ (mg/dL)	49.1	47.9	47.6	7.59	0.556	0.005	0.938
Total VFA ^7^ (mmol/L)	92.4	97.4	94.4	13.1	0.455	0.255	0.342
VFA (% of total VFA)							
Acetic acid (%)	56.7	57.3	57.9	0.839	0.151	<0.001	0.922
Propionic acid (%)	21.4	20.2	20.1	2.74	0.063	0.051	0.528
Butyric acid (%)	17.0	17.5	16.7	1.48	0.490	<0.001	0.459
Valeric acid (%)	4.86	5.05	5.20	0.570	0.551	0.301	0.545

^1^ Treatment: Control: no inclusion of *B. subtilis*; PB5700: inclusion of *B. subtilis* wild-type; and PB2OPT inclusion of *B. subtilis* engineered strain. ^2^ SE: standard error. ^3^ *p*-value of T: treatment; feed: 8 different feeds (2 different meadow hays, 2 different alfalfa hays, 2 different corn silages, and 2 different total mixed rations for lactating cows); T × Feed: interaction between T and feed. ^4^ NDFd: degradability of neutral detergent fiber expressed as percentage of the total fiber content.^5^ GP: gas production. ^6^ N-NH_3_: ammonia nitrogen concentration. ^7^ VFA: volatile fatty acids. ^abc^ Mean values in the same row with different lettering within feeds are statistically different (*p* < 0.05).

## Data Availability

All data generated or analyzed during this study are included.

## References

[1] Capper J.L., Bauman D.E. (2013). The Role of Productivity in Improving the Environmental Sustainability of Ruminant Production Systems. Annu. Rev. Anim. Biosci..

[2] Adesogan A.T., Arriola K.G., Jiang Y., Oyebade A., Paula E.M., Pech-Cervantes A.A., Romero J.J., Ferraretto L.F., Vyas D. (2019). Symposium review: Technologies for improving fiber utilization. J. Dairy Sci..

[3] Oba M., Kammes-Main K. (2023). Symposium review: Effects of carbohydrate digestion on feed intake and fuel supply. J. Dairy Sci..

[4] Donnelly D.M., de Resende L.C., Cook D.E., Atalla R.H., Combs D.K. (2018). Technical note: A comparison of alkali treatment methods to improve neutral detergent fiber digestibility of corn stover. J. Dairy Sci..

[5] Mor P., Bals B., Tyagi A.K., Teymouri F., Tyagi N., Kumar S., Bringi V., VandeHaar M. (2018). Effect of ammonia fiber expansion on the available energy content of wheat straw fed to lactating cattle and buffalo in India. J. Dairy Sci..

[6] Wang Y., McAllister T.A. (2002). Rumen Microbes, Enzymes and Feed Digestion-A Review. Asian-Australas. J. Anim. Sci..

[7] Tirado-González D.N., Miranda-Romero L.A., Ruíz-Flores A., Medina-Cuéllar S.E., Ramírez-Valverde R., Tirado-Estrada G. (2018). Meta-analysis: Effects of exogenous fibrolytic enzymes in ruminant diets. J. Appl. Anim. Res..

[8] Ferreira R.G., Azzoni A.R., Freitas S. (2021). On the production cost of lignocellulose-degrading enzymes. Biofuels Bioprod. Biorefining.

[9] Ban Y., Guan L.L. (2021). Implication and challenges of direct-fed microbial supplementation to improve ruminant production and health. J. Anim. Sci. Biotechnol..

[10] Himmel M.E., Xu Q., Luo Y., Ding S.-Y., Lamed R., Bayer E.A. (2010). Microbial enzyme systems for biomass conversion: Emerging paradigms. Biofuels.

[11] Su Y., Liu C., Fang H., Zhang D. (2020). *Bacillus subtilis*: A universal cell factory for industry, agriculture, biomaterials and medicine. Microb. Cell Factories.

[12] van Dijl J.M., Hecker M. (2013). *Bacillus subtilis*: From soil bacterium to super-secreting cell factory. Microb. Cell Factories.

[13] Earl A.M., Losick R., Kolter R. (2008). Ecology and genomics of *Bacillus subtilis*. Trends Microbiol..

[14] Koutsoumanis K., Allende A., Álvarez-Ordóñez A., Bolton D., Bover-Cid S., Chemaly M., de Cesare A., Hilbert F., Lindqvist R., EFSA Panel on Biological Hazards (BIOHAZ) (2023). Update of the list of qualified presumption of safety (QPS) recommended microorganisms intentionally added to food or feed as notified to EFSA. EFSA J..

[15] Lee N.-K., Kim W.-S., Paik H.-D. (2019). *Bacillus* strains as human probiotics: Characterization, safety, microbiome, and probiotic carrier. Food Sci. Biotechnol..

[16] Bernardeau M., Lehtinen M.J., Forssten S.D., Nurminen P. (2017). Importance of the gastrointestinal life cycle of *Bacillus* for probiotic functionality. J. Food Sci. Technol..

[17] Jha R., Das R., Oak S., Mishra P. (2020). Probiotics (Direct-Fed Microbials) in Poultry Nutrition and Their Effects on Nutrient Utilization, Growth and Laying Performance, and Gut Health: A Systematic Review. Animals.

[18] Mun D., Kyoung H., Kong M., Ryu S., Jang K.B., Baek J., Park K., Song M., Kim Y. (2021). Effects of *Bacillus*-based probiotics on growth performance, nutrient digestibility, and intestinal health of weaned pigs. J. Anim. Sci. Technol..

[19] Sun P., Wang J.-Q., Zhang H.-T. (2011). Effects of supplementation of *Bacillus subtilis* natto Na and N1 strains on rumen development in dairy calves. Anim. Feed. Sci. Technol..

[20] Sun P., Wang J.Q., Deng L.F. (2013). Effects of *Bacillus subtilis* natto on milk production, rumen fermentation and ruminal microbiome of dairy cows. Animal.

[21] Souza V.L., Lopes N.M., Zacaroni O.F., Silveira V.A., Pereira R.A.N., Freitas J.A., Almeida R., Salvati G.G.S., Pereira M.N. (2017). Lactation performance and diet digestibility of dairy cows in response to the supplementation of *Bacillus subtilis* spores. Livest. Sci..

[22] Choonkham W., Schonewille J.T., Bernard J.K., Suriyasathaporn W. (2020). Effects of on-farm supplemental feeding of probiotic *Bacillus subtilis* on milk production in lactating dairy cows under tropical conditions. J. Anim. Feed. Sci..

[23] Jia P., Tu Y., Liu Z., Li F., Yan T., Ma S., Dong L., Diao Q. (2022). Diets supplementation with *Bacillus subtilis* and *Macleaya cordata* extract improve production performance and the metabolism of energy and nitrogen, while reduce enteric methane emissions in dairy cows. Anim. Feed. Sci. Technol..

[24] Cappellozza B.I., Joergensen J.N., Copani G., Bryan K.A., Fantinati P., Bodin J.-C., Khahi M.M., DeGuzman C.N., Arriola K.G., Lima L.O. (2023). Evaluation of a *Bacillus*-based direct-fed microbial probiotic on in vitro rumen gas production and nutrient digestibility of different feedstuffs and total mixed rations. Transl. Anim. Sci..

[25] Sun P., Li J., Bu D., Nan X., Du H. (2016). Effects of *Bacillus subtilis* natto and Different Components in Culture on Rumen Fermentation and Rumen Functional Bacteria In Vitro. Curr. Microbiol..

[26] Wang Z., He Z., Beauchemin K.A., Tang S., Zhou C., Han X., Wang M., Kang J., Odongo N.E., Tan Z. (2016). Comparison of two live *Bacillus* species as feed additives for improving in vitro fermentation of cereal straws. Anim. Sci. J..

[27] Pan L., Harper K., Queiroz O., Copani G., Cappellozza B.I. (2022). Effects of a *Bacillus*-based direct-fed microbial on in vitro nutrient digestibility of forage and high-starch concentrate substrates. Transl. Anim. Sci..

[28] Dhakal R., Copani G., Cappellozza B.I., Milora N., Hansen H.H. (2023). The Effect of Direct-Fed Microbials on In-Vitro Rumen Fermentation of Grass or Maize Silage. Fermentation.

[29] Lombard V., Golaconda Ramulu H., Drula E., Coutinho P.M., Henrissat B. (2013). The carbohydrate-active enzymes database (CAZy) in 2013. Nucleic Acids Res..

[30] Krämer-Schmid M., Lund P., Weisbjerg M.R. (2016). Importance of NDF digestibility of whole crop maize silage for dry matter intake and milk production in dairy cows. Anim. Feed. Sci. Technol..

[31] Srivatsan A., Han Y., Peng J., Tehranchi A.K., Gibbs R., Wang J.D., Chen R. (2008). High-Precision, Whole-Genome Sequencing of Laboratory Strains Facilitates Genetic Studies. PLoS Genet..

[32] Doria E., Buonocore D., Marra A., Bontà V., Gazzola A., Dossena M., Verri M., Calvio C. (2022). Bacterial-Assisted Extraction of Bioactive Compounds from Cauliflower. Plants.

[33] Satlewal A., Agrawal R., Bhagia S., Das P., Ragauskas A.J. (2018). Rice straw as a feedstock for biofuels: Availability, recalcitrance, and chemical properties. Biofuels Bioprod. Biorefining.

[34] Smith J.L., Goldberg J.M., Grossman A.D. (2014). Complete Genome Sequences of *Bacillus subtilis* subsp. subtilis Laboratory Strains JH642 (AG174) and AG1839. Genome Announc..

[35] Belal E.B. (2013). Bioethanol production from rice straw residue. Braz. J. Microbiol..

[36] Krishania M., Kumar V., Sangwan R.S. (2018). Integrated approach for extraction of xylose, cellulose, lignin and silica from rice straw. Bioresour. Technol. Rep..

[37] Bergstrand M., Karlsson M.O. (2009). Handling Data Below the Limit of Quantification in Mixed Effect Models. AAPS J..

[38] Ermoli F., Bontà V., Vitali G., Calvio C. (2021). SwrA as global modulator of the two-component system DegSU in *Bacillus subtilis*. Res. Microbiol..

[39] Spanghero M., Chiaravalli M., Colombini S., Fabro C., Froldi F., Mason F., Moschini M., Sarnataro C., Schiavon S., Tagliapietra F. (2019). Rumen Inoculum Collected from Cows at Slaughter or from a Continuous Fermenter and Preserved in Warm, Refrigerated, Chilled or Freeze-Dried Environments for in Vitro Tests. Animals.

[40] Mertens D.R. (2002). Gravimetric determination of amylase-treated neutral detergent fiber in feeds with refluxing in beakers or crucibles: Collaborative study. J. AOAC Int..

[41] Pirondini M., Colombini S., Malagutti L., Rapetti L., Galassi G., Zanchi R., Crovetto G.M. (2015). Effects of a selection of additives on in vitro ruminal methanogenesis and in situ and in vivo NDF digestibility. Anim. Sci. J..

[42] Theodorou M.K., Williams B.A., Dhanoa M.S., McAllan A.B., France J. (1994). A simple gas production method using a pressure transducer to determine the fermentation kinetics of ruminant feeds. Anim. Feed. Sci. Technol..

[43] Colombini S., Rota Graziosi A., Parma P., Iriti M., Vitalini S., Sarnataro C., Spanghero M. (2021). Evaluation of dietary addition of 2 essential oils from *Achillea moschata*, or their components (bornyl acetate, camphor, and eucalyptol) on in vitro ruminal fermentation and microbial community composition. Anim. Nutr..

[44] McIntosh S., Vancov T. (2011). Optimisation of dilute alkaline pretreatment for enzymatic saccharification of wheat straw. Biomass Bioenergy.

[45] Park J.-y., Seyama T., Shiroma R., Ike M., Srichuwong S., Nagata K., Arai-Sanoh Y., Kondo M., Tokuyasu K. (2009). Efficient Recovery of Glucose and Fructose via Enzymatic Saccharification of Rice Straw with Soft Carbohydrates. Biosci. Biotechnol. Biochem..

[46] Baker J.T., Duarte M.E., Holanda D.M., Kim S.W. (2021). Friend or Foe? Impacts of Dietary Xylans, Xylooligosaccharides, and Xylanases on Intestinal Health and Growth Performance of Monogastric Animals. Animals.

